# A novel method for live imaging of human airway cilia using wheat germ agglutinin

**DOI:** 10.1038/s41598-020-71049-z

**Published:** 2020-09-02

**Authors:** Ryosuke Nakamura, Tatsuya Katsuno, Yo Kishimoto, Shinji Kaba, Masayoshi Yoshimatsu, Morimasa Kitamura, Atsushi Suehiro, Nao Hiwatashi, Masaru Yamashita, Ichiro Tateya, Koichi Omori

**Affiliations:** 1 Department of Otolaryngology-Head and Neck Surgery, Graduate School of Medicine, Kyoto University, 54 Kawahara-cho, Shogoin, Sakyo-ku, Kyoto, 606-8507 Japan; 2 Center of Anatomical, Pathological and Forensic Medical Researches, Graduate School of Medicine, Kyoto University, Kyoto, Japan; 3 Department of Otolaryngology, Kyoto-Katsura Hospital, Kyoto, Japan; 4 Department of Otolaryngology-Head and Neck Surgery, Graduate School of Medical and Dental Sciences, Kagoshima University, Kagoshima, Japan; 5 Department of Otolaryngology-Head and Neck Surgery, School of Medicine, Fujita Health University, Toyoake, Japan

**Keywords:** Fluorescence imaging, Respiratory tract diseases

## Abstract

Multiciliated epithelial cells in the airway are essential for mucociliary clearance. Their function relies on coordinated, metachronal and directional ciliary beating, appropriate mucus secretion and airway surface hydration. However, current conventional methods for observing human airway ciliary movement require ciliated cells to be detached from airway tissues. Determining the directionality of cilia is difficult. We developed a novel method to stain airway epithelial cilia to observe their movement without releasing ciliated cells. Human tracheae were obtained from patients (n = 13) who underwent laryngectomies to treat malignancies or swallowing disorders. The tracheae were treated with fluorescently labeled wheat germ agglutinin, which interacts with the acidic mucopolysaccharides present on the cilia. Epithelial surfaces were observed using an epi-fluorescence microscope equipped with a water-immersion objective lens and a high-speed camera. Ciliary movement was observable at 125 fps (13/13 samples). Ciliated cells in close proximity mostly exhibited well-coordinated ciliary beats with similar directionalities. These findings indicated that wheat germ agglutinin renders ciliary beats visible, which is valuable for observing human airway ciliary movements in situ.

## Introduction

Homeostatic conditions in the airway epithelium are maintained by mucociliary clearance, an epithelial defense mechanism. Motile cilia which cover a large part of the airway epithelium create mucous streams and contribute to the removal of extraneous particles and infectious materials^1,2^. Compromised mucociliary function may cause airway inflammation. Chronic or severe airway inflammation can lead to airway stenosis by hypertrophic subepithelial tissues or granular tissue formation. Accordingly, a genetic disorder of cilia motility has been associated with acute and chronic pulmonary infections^3,4^. Disruption in the ciliary beating direction causes sneezing- and coughing-like symptoms in mice^5^. However, the direction of ciliary beating in the human airway hasn’t been investigated. Current knowledge of human airway cilia motility is mainly based on high-speed video microscopic assessment of cells detached from the airway tissue, small pieces of the epithelial layer, and cultured cells, in which in situ directional information is lost^6,7^. Although recent developments in micro-optical coherence tomography techniques have enabled in vivo imaging of cilia movement, the resolution level is insufficient to assess directionality^8^.

The motile cilia present in airway epithelial cells are typically 0.2–0.3 µm in diameter and beat over 10 times per second^9^. Because of these properties, microscopes equipped with high-speed cameras capable of capturing > 100 images per second with a high sensitivity have been used for high-speed video microscopy. Transmitted light has conventionally been used for microscopy; therefore the system requires translucent samples, such as cells or small pieces of tissues excised from the trachea^10,11^. Alternatively, ciliary movement in thin and highly translucent mouse trachea is observable from the tracheal luminal surface using transmitted light. Kunimoto et al. successfully showed perturbation of the ciliary beating direction in an *Odf2* gene-mutated mouse^5^. However, the human trachea is sufficiently thick to obscure light, making it difficult to observe ciliary movement. Labeling cilia with fluorescent materials may be necessary to observe the movement of human airway cilia in situ.

The surface of the airway cilia is covered with a mesh-like network of membrane-tethered mucin and cell surface proteoglycans, which are considered to be important feature for maintaining appropriate physiological properties for mucociliary clearance. This implies the possibility that agglutinins could be used for labeling cilia^12,13^. MUC1 and 4 and heparan and keratan sulfate proteoglycans have been detected around airway cilia^12,14^. Sugar chains in these molecules contain sialic acid and N-acetyl glucosamine, which can be a target of wheat germ agglutinin (WGA)^15–19^. Okada et al. have shown that WGA can be attached to the cilia of cultured airway epithelial cells. This strongly suggests the potential usefulness of WGA for airway cilia visualization^14^.

In this study, we found that WGA) attaches to the surface of airway epithelial cilia in murine and human tracheae. WGA conjugated with a fluorescent dye enabled live imaging of airway epithelial cilia. Herein, we report a method for live imaging of cilia, and show aberrant ciliary movement in 2 tracheal samples with abnormal cilia, obtained from patients who underwent radiation therapy and tracheotomy.

## Results

### Binding capacity of WGA to airway cilia

**Figure 1:**
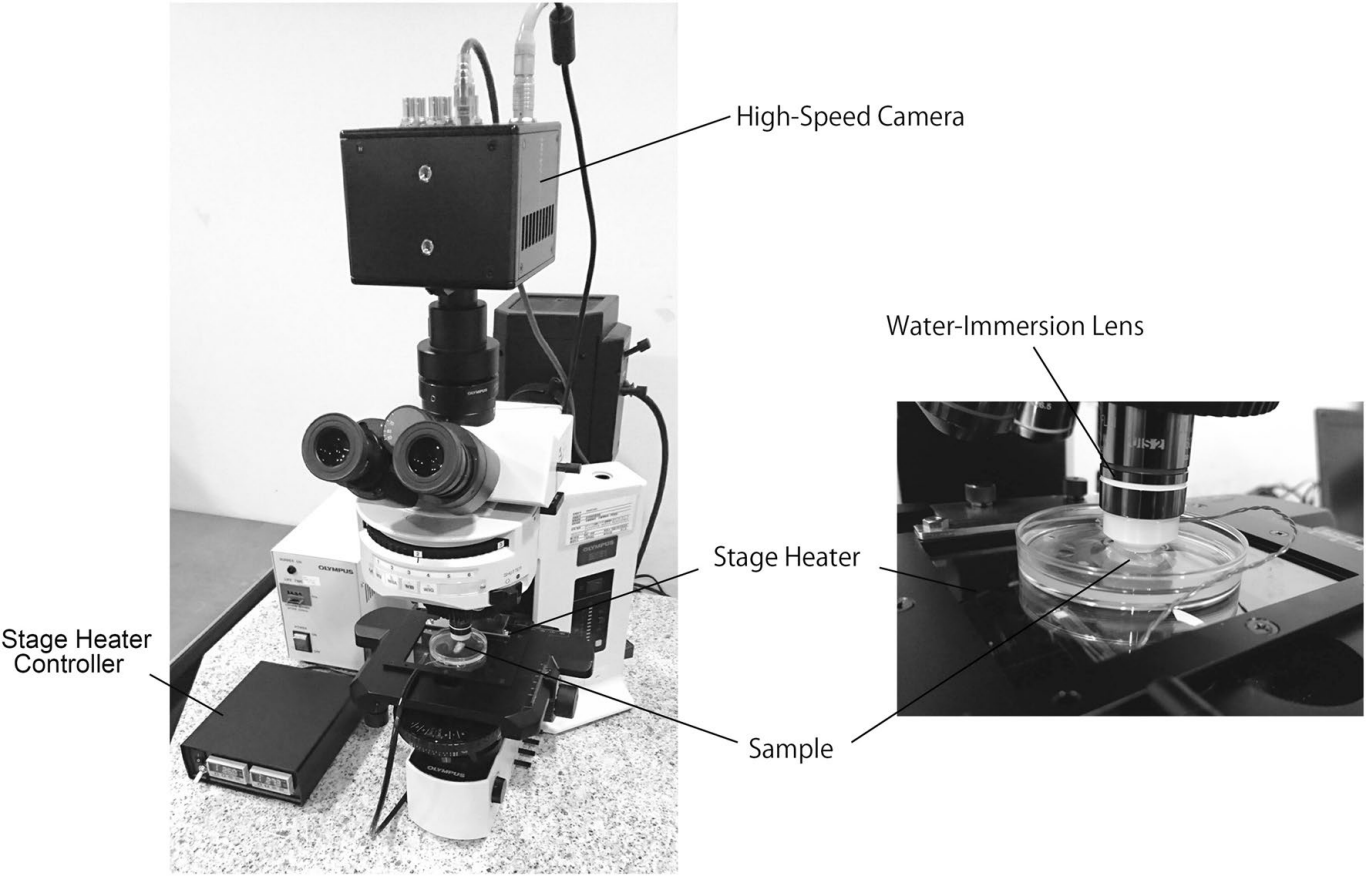
Instruments used for observation of airway epithelial ciliary movement.

We initially used mouse tracheae to determine the capability of WGA to stain the cilia of living airway epithelial cells. Since mouse tracheae are sufficiently thin so as to observe ciliary movement through transmitted light, live images of WGA-stained cilia can be compared with those obtained using transmitted light. An epifluorescence microscope equipped with a × 60 water-immersion lens and a high-speed camera were used for observation (Fig. 1).

**Figure 2:**
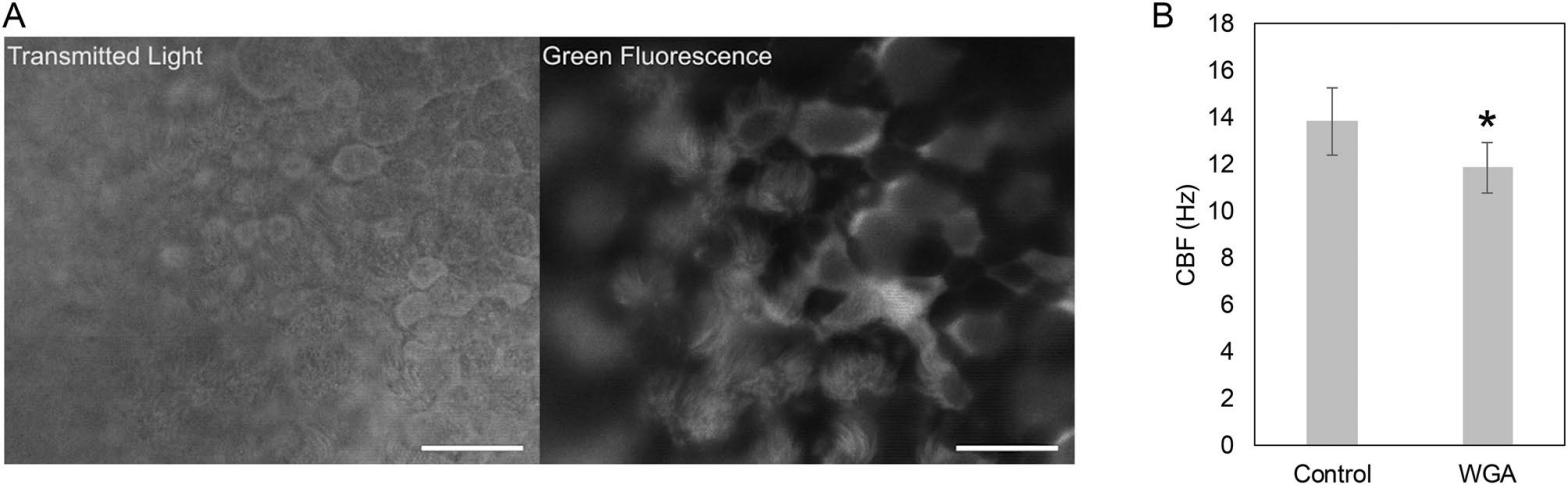
Viable ciliated cells in mouse trachea stained with FITC-WGA. Captured movie frames of ciliary movement (**A**; supporting information Movie 1). Images were captured via a transmitted light (left) and epi-luminescent light (right). Bar: 20 µm. Ciliary beat frequency measured in control and WGA-stained tracheae (**B**). Data are shown as mean ± SD (n = 5). Statistical significance is denoted by an asterisk (Student’s *t*-test, *P* < 0.05).

Tracheal epithelial cilia were successfully visualized with fluorescein isothiocyanate-WGA (FITC-WGA) in 5 out of 5 mice (Fig. 2A, Movie 1). A large proportion of the cilia observed by transmitted light were positive for FITC-WGA, and the movements of cilia stained with FITC-WGA were similar to those observed via transmitted light. The movement of FITC-WGA-stained cilia was observed as a directional beat of hair-like shapes. The timing of ciliary beat initiation differed in ciliated cells in close proximity to each other. In addition, epithelia stained with FITC-WGA still exhibited the capacity to transport fluorescent micro-beads (data not shown). These results suggested that metachronal ciliary beating and transport functions are maintained even after FITC-WGA treatment. On the other hand, ciliary beat frequency (CBF) in the FTC-WGA-treated epithelium was approximately 15% lower than that in untreated control epithelium (Fig. 2B). It may be possible that the aggregation of carbohydrates and WGA hampers the ciliary beat, at least to some extent.

### Visualization of human airway cilia movement using fluorescence-labeled WGA

**Figure 3:**
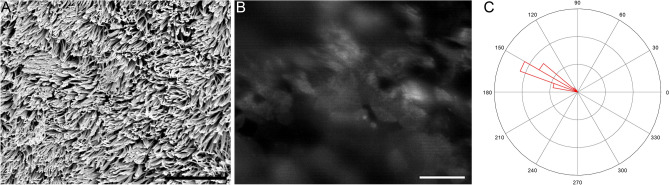
Undamaged human tracheal epithelial cilia. SEM image (**A**), and a captured movie frame of FITC-WGA-stained ciliary movement (**B**; see Movie 2 for better understanding of ciliary movement). Bars indicate 10 µm (**A**) and 20 µm (**B**). The distribution of ciliary beat directionality is shown as a rose diagram (**C**).

FITC-WGA staining also enabled the observation of ciliary movement in all 13 human tracheal samples. A representative movie of the ciliary movement is shown in Movie 2, and the captured frame of Movie 2 and scanning electron microscopy (SEM) image of the same sample are shown in Fig. 3. The mean CBF of ciliated cells in the tracheal sample was approximately 12 Hz. The directionality of the ciliary beating was discernible. Therefore, these data suggested that fluorescence-labeled WGA is useful for observing rapid ciliary movements and for analyzing their speed and directionality in human airway samples.

### Tracheal epithelial ciliary movement in patients who underwent radiation therapy and tracheotomy

**Figure 4:**
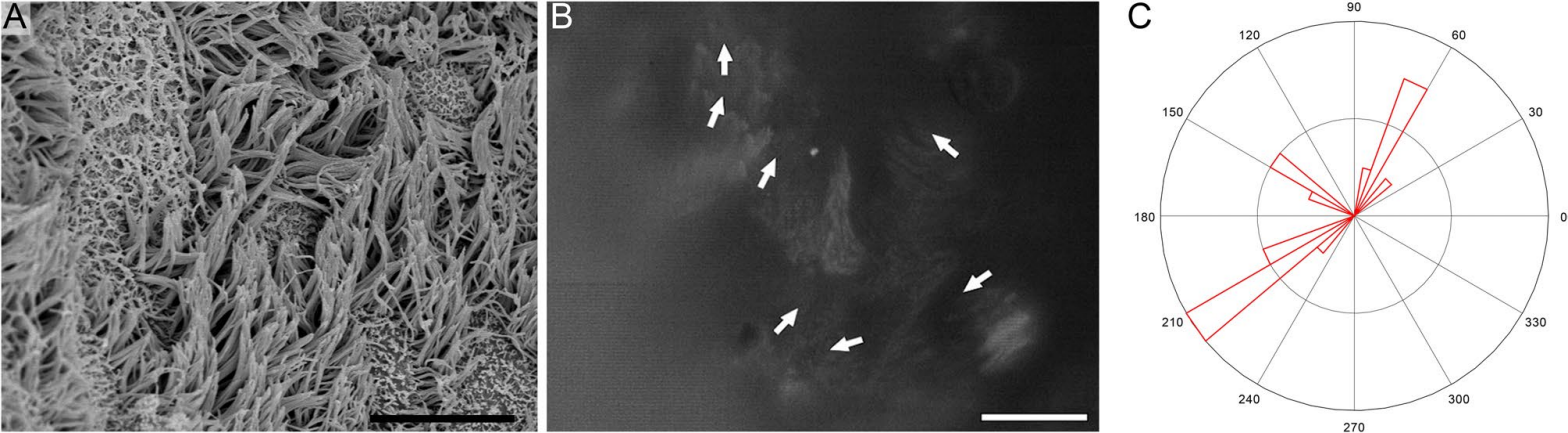
Cilia in a human trachea obtained from a patient who previously underwent radiation therapy. SEM image (**A**), and a captured movie frame of FITC-WGA-stained ciliary movement (**B**; see Movie 3 for better understanding of ciliary movement). Bars indicate 10 µm (**A**) and 20 µm (**B**). The distribution of ciliary beat directionality is shown as a rose diagram (**C**).

A tracheal sample, obtained from a patient who had been irradiated for cancer treatment, had damage to the epithelium. Some regions consisted of ciliated cells with normal and abnormally short cilia (Fig. 4). The ciliary beat in these regions was not unidirectional, and ciliated cells with different directionalities were observed (Movie 3). To evaluate the disorganization of directionality, the standard deviation of the ciliary beat direction was determined. The standard deviation in the irradiated sample was higher than that in the normal epithelium (1.29 vs. 0.07).

**Figure 5:**
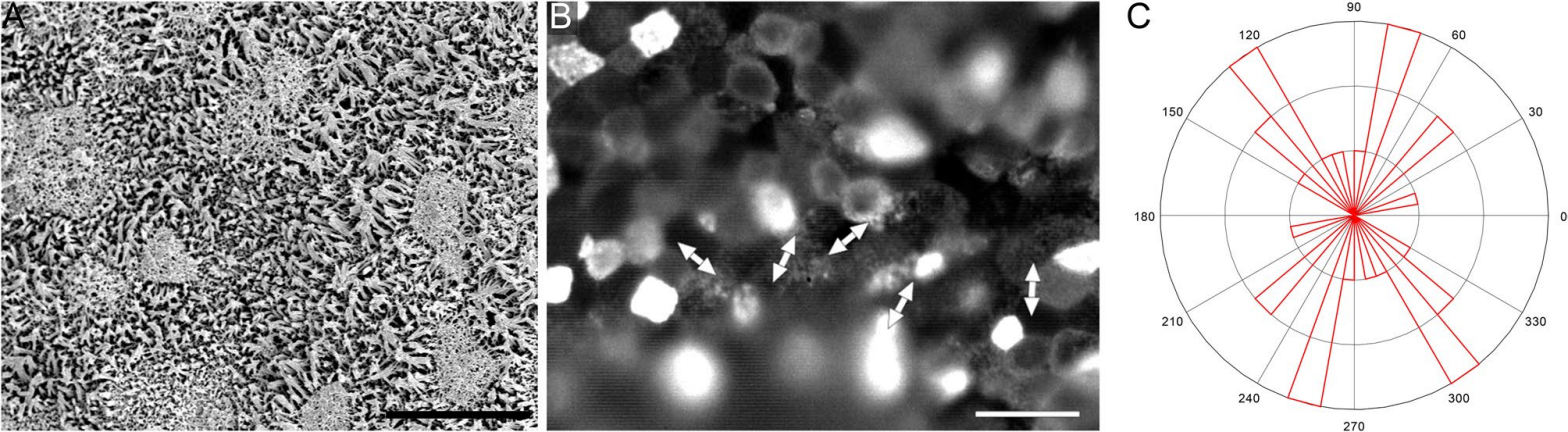
Cilia in human trachea obtained from a patient who previously received tracheotomy. SEM image (**A**), and a captured movie frame of FITC-WGA-stained ciliary movement (**B**; see Movie 4 for better understanding of ciliary movement). Bars indicate 10 µm (**A**) and 20 µm (**B**). The distribution of the ciliary beat axis is shown as a rose diagram (C).

A tracheal sample was obtained from a patient who underwent tracheotomy approximately 1 month prior to sampling. In some regions, the epithelium was mainly covered by short cilia (Fig. 5). Due to the shortness of the cilia, it was unclear where the power stroke of cilia was directed; however, the axis of the cilium stroke appeared to be disorganized.

These results suggested that using fluorescence-labeled WGA may reveal dysfunctions in ciliary directionality after airway epithelial damage.

## Discussion

The current study showed FITC-WGA enabled observation of the human tracheal ciliary beat, and to discern the directionality or axis of such beating in normal and damaged epithelia. Recent studies have shown that the directionality of airway ciliated cells is regulated by planar cell polarity (PCP) signaling. PCP is a property that coordinates the patterning of cells and the orientation of their structures along tissue planes^20^. In each ciliated airway cell, the distal-to-proximal ciliary movement is organized by the proximal localization of Vangl, and the distal localization of Frizzled^21,22^. However, the mechanisms regulating tissue-level planar polarity in the airway epithelium are largely unknown. Moreover, re-organization and maintenance of PCP during regeneration and cellular turn-over processes of the airway epithelium have rarely been investigated. Our method may be useful for providing further insights into PCP regulation in the airway epithelium.

It may be beneficial to recognize epithelial condition by FITC-WGA staining. In the airway, epithelial stem/progenitor cells exhibit great proliferation capacity after damage^23,24^. The morphological properties of regenerated airway epithelia are similar to those of undamaged epithelia, except in certain chronic respiratory diseases. However, it is largely unexplored whether ciliary beating is restored after the regeneration process^24,25^. Our method can locate damaged regions, and analyze CBF and directionality in situ.

Mouse tracheae stained with FITC-WGA exhibited lower CBF than unstained ones. Hence, CBF measured in FITC-WGA-stained samples was not comparable with unstained samples. It should also be noted that the sensory function of airway cilia may be hampered by WGA interactions. It has been reported that airway cilia are chemosensory, and WGA reduces the responses of olfactory cilia to odorants^26,27^.

In summary, fluorescence-labeled WGA bound to the surface of human airway epithelial cilia. The movement of fluorescence-labeled airway cilia was captured using a high-speed camera at 125 fps. Additionally, fluorescence-labeling rendered the directionality of ciliary beating discernible. Therefore, using fluorescence-labeled WGA may be useful for analyzing the movement of human airway epithelial cilia.

## Methods

### Mouse trachea

Five mice (C57BL/6 strain, 12 weeks old, female) were euthanized with approval from the Animal Care and Use Committee of Kyoto University by cervical dislocation, and tracheae were obtained. All animal experiments were performed in accordance with the institutional Guidelines for Animal Experiments, the Japanese Government Law Concerning the Protection and Control of Animals, and the Japanese Government Notification on Feeding and Safekeeping of Animals.

### Human trachea

Human tracheae were obtained from 13 patients who underwent laryngectomy for treatment of dysphagia, malignant tumors in the pharynx, larynx, esophagus and tongue, and subglottic stenosis. Before laryngectomy, 4 and 4 of patients received cervical radiation, and tracheotomy within 2 years, respectively. Written informed consent were obtained from all the patients before the surgery, and this study was approved by the Kyoto University Hospital Ethics Community (R1204-2) and conducted in accordance with the Kyoto University Hospital Institutional Guidelines.

### Fluorescent labeling of tracheae

Tracheae were cut into strips, and then attached to plastic dishes using Vetbond (3 M, Maplewood, MN, USA) so that their luminal sides were facing upward. After rinsing with Dulbecco’s modified Eagle’s medium (DMEM) -high glucose (Thermo Fisher Scientific, Waltham, MA, USA), the specimens were incubated in DMEM-high glucose containing 8 µg/mL FITC-WGA (Vector Laboratories, Burlingame, CA, USA) for 1 h. Unattached FITC-WGA was removed by rinsing with DMEM-high glucose.

### Observation of cilia movement

An upright microscope (BX-51; Olympus, Tokyo, Japan), equipped with a × 60 water-immersion objective lens (Olympus), and a high-speed camera (FASTCAM mini UX50; Photron, Tokyo, Japan), was used to observe ciliary movement (Fig. 1). Mouse and human tracheae stained with FITC-WGA were placed in phenol red-free DMEM/F-12 (Nacalai Tesque, Kyoto, Japan), and the luminal surfaces of the tracheae were observed through transmitted light and/or epi-fluorescent illumination. Images of ciliary movement were captured using a high-speed camera at frame rates of 50 or 125 fps.

### Analysis of ciliary beat frequency

Ciliary movement in human trachea was recorded at a frequency of 125 fps. The mean CBF in the recorded region was analyzed using the ImageJ plugin ciliaFA (National Institutes of Health, Bethesda, MD, USA)^28^. Statistical differences between CBF in unstained and FITC-WGA-stained mouse tracheae were analyzed by Student’s *t-*test. *P* < 0.05 was considered significantly different.

### Analysis of the ciliary beat direction and axis

Directions and axes of ciliary movement in each ciliated cell were measured using ImageJ. Measurements were performed for at least 15 ciliated cells in one field of view (approximately 210 × 170 µm). Rose diagrams were created using Octave (https://www.gnu.org/software/octave/) to show the distributions of directions and axes. The standard deviation of the ciliary beat direction was determined using the following equations^29^:

1 $$v= \mathrm{cos}\theta +i\mathrm{sin}\theta$$

2 $$R=\mathrm{abs }(\frac{1}{N}\sum_{j}{v}_{j}) (j=1, 2, 3,\dots , N)$$

3 $$S= \sqrt{-2\mathrm{ln}R}$$

where ciliary beat directions were regarded as unit vectors, *v* is the unit vector in the complex plane, θ is the ciliary beat direction, *i* is the imaginary unit, *R* is the length of the mean of unit vectors, *N* is the number of measured cells, and *S* is the standard deviation.

### Scanning electron microscopy

After observation of ciliary movement, human tracheae were pre-fixed with 2% (w/v) formaldehyde and 2.5% (w/v) glutaraldehyde in 100-mM HEPES [4-(2-hydroxyethyl)-1-piperazineethanesulfonic acid) buffer for 24 h at 4 °C and post-fixed using 1% osmium tetroxide. The fixed samples were dehydrated using increasing concentrations of ethanol (50%, 60%, 70%, 80%, 90%, 99%, and 100%). Ethanol was substituted with tert-butyl alcohol and then samples were freeze-dried. The samples were sputter-coated with platinum-palladium alloy using an ion coater (IB-3; Eiko, Tokyo, Japan) and the luminal surfaces were observed using a scanning electron microscope (S-4700; Hitachi, Tokyo, Japan).

## Supplementary information

Supplementary Legends

Supplementary Movie 1.

Supplementary Movie 2.

Supplementary Movie 3.

Supplementary Movie 4.

## Data Availability

All of the data in this study can be obtained from corresponding author upon reasonable request.
