# Supplementary material for: A novel method for live imaging of human airway cilia using wheat germ agglutinin
Source: Sci Rep. 2020 Sep 2;10:14417. doi: 10.1038/s41598-020-71049-z (PMC7468155; doi:10.1038/s41598-020-71049-z)
Supplement: Supplementary file 1 — Supplementary Legends [file 41598_2020_71049_MOESM1_ESM.docx]

**A novel method for live imaging of human airway cilia using wheat germ agglutinin.**

Ryosuke Nakamura, PhD^1^

Tatsuya Katsuno, PhD^2^

Yo Kishimoto, MD, PhD^1*^

Shinji Kaba, MD^1^

Masayoshi Yoshimatsu, MD^1^

Morimasa Kitamura, MD, PhD^1^

Atsushi Suehiro, MD, PhD^1^

Nao Hiwatashi, MD, PhD^3^

Masaru Yamashita, MD, PhD^4^

Ichiro Tateya, MD, PhD^5^

Koichi Omori, MD, PhD^1^

1: Department of Otolaryngology–Head and Neck Surgery, Graduate School of Medicine, Kyoto University, 54 Kawahara-cho, Shogoin, Sakyo-ku, Kyoto, 606-8507 Japan

2: Center of Anatomical, Pathological and Forensic Medical Researches, Graduate School of Medicine, Kyoto University, Kyoto, Japan

3: Department of Otolaryngology, Kyoto-Katsura Hospital, Kyoto, Japan

4: Department of Otolaryngology-Head and Neck Surgery, Graduate School of Medical and Dental Sciences, Kagoshima University, Kagoshima, Japan

5: Department of Otolaryngology-Head and Neck Surgery, School of Medicine, Fujita Health University, Toyoake, Japan

*Correspondence and requests for reprints should be addressed to Yo Kishimoto, MD, PhD

Department of Otolaryngology–Head and Neck Surgery, Graduate School of Medicine, Kyoto University

54 Kawahara-cho, Shogoin, Sakyo-ku, Kyoto, 606-8507 JAPAN.

E-mail: [y_kishimoto@ent.kuhp.kyoto-u.ac.jp](mailto:y_kishimoto@ent.kuhp.kyoto-u.ac.jp)

**Movie 1. Ciliary movement in the mouse trachea stained with FITC-WGA.**

Unfixed mouse trachea stained with FITC-WGA and observed through transmitted and epi-fluorescent lights. Playback speed: 0.24×. Bar: 20 µm.

**Movie 2. Ciliary movement in an undamaged human tracheal epithelium.**

Unfixed human trachea stained with FITC-WGA and observed through transmitted and epi-fluorescent lights. Playback speed: 0.24×. Bar: 20 µm.

**Movie 3. Ciliary movement in a human trachea obtained from patients who previously underwent radiation therapy.**

Unfixed human trachea stained with FITC-WGA and observed through transmitted and epi-fluorescent lights. Playback speed: 0.6×. Bar: 20 µm.

**Movie 4. Ciliary movement in a human trachea obtained from patients who previously received tracheotomy.**

Unfixed human trachea stained with FITC-WGA and observed through transmitted and epi-fluorescent lights. Playback speed: 0.3×. Bar: 20 µm.
